# Successful application of vagus nerve stimulation in super refractory status epilepticus associated with MERRF syndrome

**DOI:** 10.1016/j.ebr.2025.100769

**Published:** 2025-04-14

**Authors:** Leyla Baysal, Sabrina Jobi, Simone Zimmermann, Ann-Kristin Helmers, Nils Gerd Margraf

**Affiliations:** a Epilepsy Center, Department of Neurology, University Hospital Schleswig-Holstein, Campus Kiel, Arnold-Heller-Str. 3, 24105 Kiel, Germany; b Department of Neurosurgery, University Hospital Schleswig-Holstein, Campus Kiel, Arnold-Heller-Str. 3, 24105 Kiel, Germany

**Keywords:** Super-refractory status epilepticus, Vagus nerve stimulation, Mitochondrial disease, MERRF syndrome

## Abstract

Myoclonic epilepsy with ragged red fibers (MERRF) syndrome is a rare mitochondrial disorder marked by myoclonus, ataxia, cognitive impairment, myopathy, and sensorineural hearing loss. Seizures in MERRF syndrome are often resistant to standard antiseizure medication. We present a unique case of an adult patient with MERRF syndrome, caused by the m.8344A > G point mutation, who developed super-refractory status epilepticus (SRSE), successfully managed by the initiation and rapid escalation of vagus nerve stimulation (VNS) therapy. Over a two-year follow-up period, the VNS therapy enabled a long-term control of generalized tonic-clonic seizures. A further status epilepticus has not yet occurred. VNS may therefore be a promising therapeutic option for managing SRSE in patients with mitochondrial epilepsy syndromes.

## Highlights

• VNS implantation during SRSE can stop seizures and prevent recurrence effectively.
• VNS may be a promising therapy for SRSE in mitochondrial epilepsy syndromes.
• Rapid VNS titration may enhance outcomes by accelerating efficacy without major side effects.

## 1 Introduction

Refractory status epilepticus (RSE) and super-refractory status epilepticus (SRSE) are severe neurological emergencies with high morbidity and mortality [1]. These conditions can result in permanent neuronal damage, alterations in brain networks, and long-term disability. Treatment of RSE/SRSE often involves first- and second-line antiseizure medications (ASMs), anesthetics, and, in some cases, alternative interventions like ketogenic diet, therapeutic hypothermia, brain surgery, immune therapy or neuromodulation methods when standard therapies fail [2]. Vagus nerve stimulation (VNS) has been recognized for its potential to reduce seizure frequency, but its efficacy in RSE/SRSE remains under investigation [[3], [4], [5], [6]]. Myoclonic epilepsy with ragged red fibers (MERRF) is a multisystem mitochondrial disorder characterized by myoclonus, cerebellar ataxia, and ragged-red fibers in muscle tissue [7]. This report presents the successful use of VNS with rapid titration in a patient with SRSE associated with MERRF syndrome, caused by the m.8344A > G point mutation.

## 2 Case Presentation

A 38-year-old female with a known diagnosis of MERRF syndrome, due to the m.8344A > G mitochondrial DNA mutation, was admitted because of a second episode of convulsive status epilepticus (SE) during the month prior to admission. At the time of admission, she was being treated with clonazepam 2.5 mg/d, lacosamide 300 mg/d, perampanel 6 mg/day, brivaracetam 100 mg/d, coenzyme Q 10 100 mg/d, thiamine 100 mg/d, levocarnitine 1000 mg/d. No obvious triggers for the recurrent SE including medication non-adherence were identified.

### 2.1 Previous history of the disease

The patient had an uncomplicated birth and normal neurodevelopment with no family history of epilepsy. However, her maternal aunt had mild muscle weakness and developmental delay with no confirmed neuromuscular diagnosis. At age 14, the patient developed progressive myoclonic jerks, mainly in the lower limbs, which worsened over time and were accompanied by fatigue. At the age of 16, she had her first unprovoked bilateral tonic-clonic seizure in a nightclub, leading to a suspected diagnosis of juvenile myoclonic epilepsy (JME).

For 2.5 years, seizure control was achieved with valproate, however, discontinuation due to future pregnancy considerations resulted in a bilateral tonic-clonic seizure. Transition to lamotrigine exacerbated the myoclonus, necessitating a switch to levetiracetam, which provided partial symptom relief, but daily myoclonus episodes persisted. These together with absence-like seizures significantly impairing her quality of life. Cognitive function remained intact initially, and she pursued a degree in technical mathematics. By age 26, progressive fatigue, muscle weakness, ataxia, and dysarthria prompted further diagnostic evaluation. Extensive testing, including brain magnetic resonance imaging (MRI), laboratory studies, and lumbar puncture, was unremarkable. Subsequent genetic analysis confirmed the MERRF syndrome (m.8344A > G mutation) with additional assessments excluding other mitochondrial disorders. EEG recordings performed during breakthrough seizures ten years after the diagnosis showed moderate to severe encephalopathy with multifocal epileptogenic activity across multiple cortical regions.

Despite numerous attempts to control her seizures using gradual dose escalation and polytherapy with levetiracetam, zonisamide, clobazam, and topiramate her condition progressively worsened. Over the four years preceding her most recent admission she experienced eight episodes of SE, primarily triggered by deliberate dose reductions of the medications due to their side effects. These episodes included convulsive SE, myoclonic SE, and nonconvulsive SE, necessitating multiple intensive care admissions with burst-suppression anesthesia using propofol, isoflurane, midazolam, and sufentanil. ASMs were repeatedly adjusted, incorporating additional agents such as perampanel, lacosamide, clonazepam, brivaracetam, and lorazepam as needed. Despite persistent intermittent seizures of varying semiologies and pharmacoresistance, she maintained good cognitive function, remained independent in a wheelchair, and had a good quality of life with intensive family support.

### 2.2 Hospital follow-up

**Fig. 1:**
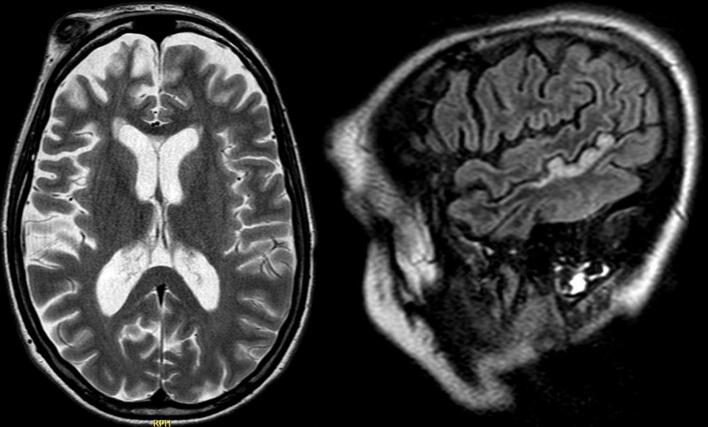
Brain MRI showed global frontal atrophy and T2 hyperintensities in the right MCA territory, consistent with postictal changes, with no new structural abnormalities.

Upon hospital admission, the patient was intubated, sedated, and ventilated because of the convulsive SE and the failure to respond to conventional ASMs (intravenous (IV) lorazepam, levetiracetam, lacosamide) and anesthetic agents (midazolam, propofol). She was transferred to the intensive care unit (ICU) for burst-suppression therapy with propofol, then isoflurane and sufentanil, requiring volume resuscitation and vasopressors. Laboratory results showed elevated inflammatory markers attributed to aspiration pneumonia, which improved with intravenous antibiotics. Initial cerebral computer tomography (CT) was unremarkable. Brain MRI revealed global frontal atrophy and T2-weighted signal hyperintensities in the right middle cerebral artery territory consistent with postictal changes, with no new structural abnormalities compared to previous imaging (Fig. 1).

**Fig. 2:**
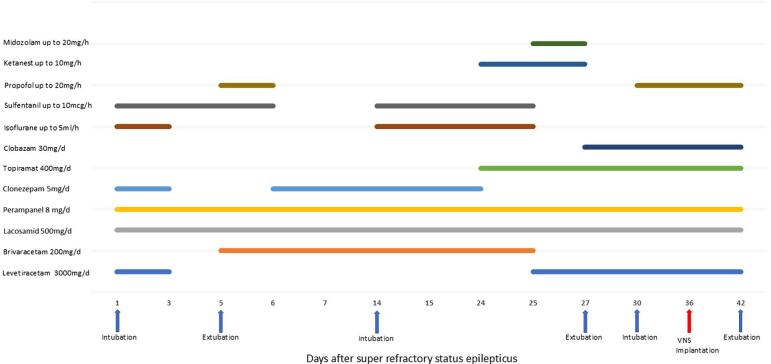
Treatment at hospital.

**Fig. 3:**
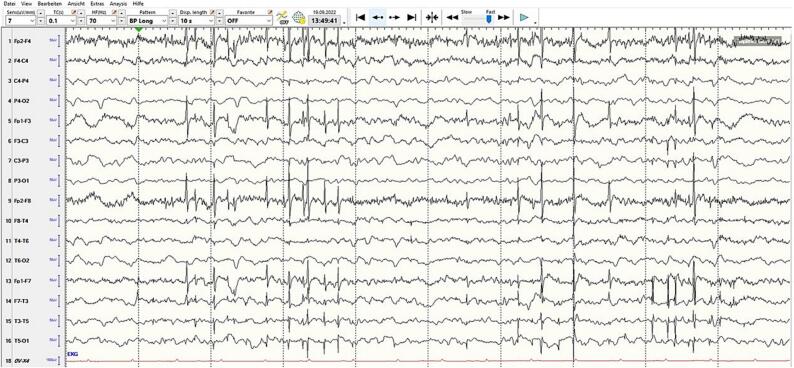
The EEG indicates moderate encephalopathy with bifrontally accentuated, multifocal, and generalized epileptiform potentials, plus artifacts linked to periorbital-dominant myoclonus.

Sedation was gradually reduced, and the patient was extubated five days after admission. Non-invasive ventilation was initiated, later reduced to high-flow oxygen, and finally oxygen via nasal cannula as her alertness improved. ASM therapy (clonazepam, brivaracetam, perampanel, lacosamide) continued with dose adjustments. However, due to worsening breakthrough focal seizures and generalized multifocal myoclonus with loss of consciousness, she was readmitted to the ICU after nine days. Burst suppression was re-induced for ten days using isoflurane, midazolam, propofol, sufentanil, and ketamine, with levetiracetam, topiramate, and clobazam added to the regimen (Fig. 2). Her respiratory status worsened after another extubation attempt. This led to renewed SE requiring reintubation. The seizures were managed with low-dose propofol. Her husband refused consent for a percutaneous endoscopic gastrostomy (PEG) insertion or tracheostomy in accordance with the patient's previously expressed wishes. A series of routine EEGs (10–30 min in duration) performed following the discontinuation of sedation revealed evidence of moderate encephalopathy with generalized, bifrontally accentuated, partially multifocal and generalized epileptiform potentials, along with EEG artifacts corresponding to videographically documented periorbital-dominant myoclonus (Fig. 3).

On the 36th day of hospitalization, a VNS device (VNS Therapy system, Sentiva M1000 Generator, LivaNova USA, Inc., Houston, TX) was implanted, and VNS was activated on the day of implantation. The output current was rapidly increased (range 0.125–0.5 mA/24 h) until peak amplitudes of 2 mA were achieved over six days (Table 1). The final parameters were: intensity 2 mA, 30 sec on, 1.8 min off, pulse width 250 μs, frequency 20 Hz with a duty cycle of 25 %. Over the course of a week, the propofol dose was gradually tapered and eventually discontinued, leading to improved spontaneous breathing and alertness. Following the rapid titration of VNS intensity to 2 mA, the patient exhibited significant clinical improvement with reduced myoclonus activity and was successfully extubated seven days after VNS implantation. After extubation she continued to have daily generalized and multifocal myoclonus, particularly affecting her left arm, head, and face. This was partially improved with intermittent IV lorazepam and dexmedetomidine administration. She then experienced two focal to bilateral tonic-clonic seizures within the first three weeks after VNS implantation, possibly triggered by the reduction of the dexmedetomidine infusion and a systemic infection. Our patient showed improvement of her myoclonic jerks starting from day 7 post-implantation and was free from generalized tonic-clonic seizures by day 21 post-implantation. Her follow-up EEGs showed no evidence of an SE pattern but revealed moderate to severe diffuse encephalopathy and lateralized epileptogenic activity in the right hemisphere. During the follow-up, alertness, orientation and swallowing dysfunction improved significantly and myoclonus appeared less frequently**.** The ASMs were adjusted numerous times, and she was discharged 45 days after VNS implantation and 83 days after admission on a regimen of levetiracetam (3000 mg/day), lacosamide (600 mg/day), perampanel (6 mg/day), topiramate (250 mg/day) and lorazepam (4 mg/day) with a planned reduction strategy. Upon discharge, ongoing outpatient speech and swallowing therapy, as well as physiotherapy were recommended. Two weeks after discharge, she was able to swim alone using a swimming mask in the pool at her house (Video 1).

**Table 1:** Scheme of rapid increase of VNS intensity.

	1.day	2.day	3.day	4.day	5.day	6.day
Current output (mA)	0,125 0,25 0,375 0,5	0,625	0,75 0,875 1	1,125 1,25 1,375	1,5 1,75	2
Signal Frequency (Hz)	20	20	20	20	20	20
Pulse width (msec)	250	250	250	250	250	250
Signal on time (sec)	30	30	30	30	30	30
Signal off time (min)	5	5	5	5	5	1,8
Duty cycle	10	10	10	10	25	25

Days after implantation of vagal nerve stimulator.

Over the two-year follow-up period, the patient experienced no recurrence of bilateral tonic-clonic seizures or SE. Her myoclonus activity decreased but persisted. Focal seizures with impairment of consciousness appeared to be very rare. She is permanently confined to a wheelchair and receives support from her husband and family for physical care and mobilization**.** The patient continued to receive regular outpatient physiotherapy, speech therapy and occupational therapy, from which she clearly benefited. During the first two months of her VNS therapy she reported hoarseness, recurrent episodes of coughing fits and episodes of asymptomatic bradycardia recorded from the stimulator. These symptoms improved over time. A long-term ECG showed no relevant findings. No other adverse effects of VNS were reported. Her quality of life improved, with continued monitoring and adjustments to her ASMs as necessary. Eighteen months after the last SE, a video EEG monitoring was performed, which showed no signs of seizure or interictal epileptiform activity but indicating moderate encephalopathy**.** Cognitive testing revealed deficits in verbal and nonverbal memory, as well as impairments in working memory. However, her verbal communication abilities improved after speech therapy.

## 3 Discussion

This case report highlights the successful application of VNS therapy with rapid titration in a patient with SRSE and a primary mitochondrial disease. The successful management of the recurrent, life-threatening convulsive SE with the aid of VNS was remarkable, and resulted in a substantial improvement in the patient’s quality of life. VNS therapy appeared to have long-term effectiveness in achieving freedom from generalized tonic-clonic seizures and reduction of myoclonus and focal impaired awareness seizures as well as further SE.

In a study in adults with genetically confirmed mitochondrial disorders the overall prevalence of epilepsy was 23.1 % [8]. The prevalence was 34.9 % for the m.3243A > G genotype (mitochondrial encephalopathy, lactic acidosis, and stroke-like episodes-MELAS), and 92.3 % of the patients with the m.8344A > G mutation had seizures associated with MERRF [8]. The clinical features of MERRF include myoclonus, epilepsy, ataxia, cognitive decline, myopathy, sensorineural hearing loss, pigmentary retinopathy, and multiple symmetric lipomatosis [9]. Myoclonic jerks were seen in all patients with m.8344A > G mutation, while focal seizures were more common in other genotypes [8]. In an Italian registry of patients with mitochondrial disorders, SE was reported in two out of 24 cases (8 %) [10]. The exact mechanisms behind mitochondrial SE are not completely understood and may involve disruptions in energy metabolism, oxidative stress, immune system dysfunction, and altered mitochondrial behavior [9].

Managing seizures in patients with mitochondrial disorders is difficult, as certain ASMs, such as,e.g. sodium valproate (valproic acid, VPA) are known to cause mitochondrial toxicity in POLG disease [11]. Treatment approaches for mitochondrial SE have included ASMs, anesthetic drugs, magnesium supplementation, high-dose steroids, immunoglobulin therapy, VNS, and surgical methods and have had varying degrees of success [9]. In previous case series VNS had no effect or an unspecified effect in controlling intractable seizures in a group of children with pharmacoresistant epilepsy and mitochondrial disease [[12], [13], [14]]. There is also anecdotal evidence that VNS devices have been successful in improving seizure control in patients with mitochondrial disease [15,16,17]. Limited evidence, including our case, suggests that rapid VNS titration may be effective in treating SE in patients with MERRF, other progressive myoclonic epilepsies (PMEs), and genetic epilepsies [5,[17], [18], [19]]. Given that PME is a progressive condition, the efficacy of VNS may diminish over time [20,21]. The case report is based on retrospective hospital records, which limits the ability to quantify seizure frequency and the detailed use of rescue medications. Given the marked improvement in seizure control post-implantation, we believe that VNS contributed significantly to the patient’s clinical condition.

While VNS is a well-established treatment for refractory epilepsy, it remains unclear whether its immediate use in a patient with SE can effectively end the seizure episode [4]. A 2019 systematic review on the effectiveness of VNS in RSE and SRSE found that acute VNS implantation led to seizure cessation in 74 % (28/38) of the patients. However, 18 % (7/38) of the patients did not respond, and four deaths occurred (11 %), all attributed to the underlying condition and not to the VNS implantation. The authors noted that the analysis included many case reports, which could lead to reporting bias and potentially inflated results, as negative outcomes are less likely to be published [4]. In line with other studies, our case report demonstrates that rapid titration of VNS not only leads to the cessation of SE but also allows the depth of anesthesia to be reduced, the assessment of the patient's level of consciousness and neurological status, and long-term seizure control [6,22,23]. Further clinical experience is essential to assess the true efficacy of this treatment in clinical practice. While VNS in the setting of RSE or SRSE is not a novel application, the novelty of this case lies in the rapid titration of VNS and its use in a patient with PME, a less commonly explored context for this treatment approach. We believe this underscores the potential of VNS in managing complex and refractory epilepsy syndromes.

The mechanism of chronic VNS involves electrical stimulation of the left vagus nerve, which activates the nucleus of the tractus solitarius (NTS) and results in the release of norepinephrine from the locus coeruleus (LC) and serotonin from the raphe nuclei (RN). These neuromodulators produce anticonvulsant effects leading to enhanced inhibition and decreased excitability, alterations in cerebral blood flow, disruption of pathological neuronal synchronization, and anti-inflammatory actions mediated by norepinephrine [24]. EEG studies suggest that VNS acutely desynchronizes both interictal and ictal rhythms, potentially preventing hypersynchronous activity and inhibiting the propagation of focal-onset seizures [25,26]. A single-photon emission computed tomography (SPECT) study in a group of patients with drug-resistant focal epilepsy, conducted before and one year after VNS implantation, showed that the clinical effectiveness of VNS was associated with the upregulation of GABA-A receptor density [27]. This upregulation may have potential implications for the treatment of SE, particularly in modulating epileptogenesis and improving therapeutic outcomes. Rapid titration of VNS may optimize patient outcomes by achieving a more rapid onset of therapeutic efficacy without a substantial increase in adverse effects [28,29]. The rapid titration of VNS in this patient did not lead to significant acute side effects, as the patient was under sedation during the initial implantation and titration. Follow-up side effects, including hoarseness, coughing fits, and bradycardia, were mild, did not impact the patient's quality of life, and resolved without requiring adjustments to the VNS settings.

Our findings are consistent with previous reports suggesting that VNS implantation during SRSE can effectively terminate seizures and prevent recurrence. The acute and well-tolerated use of VNS in this context offers a valuable alternative when standard ASMs and anesthetics fail, and surgical options are either unavailable or inappropriate. Additionally, this case demonstrates the potential for VNS to reduce the risk of SE relapse in patients with genetic epilepsy, contributing to long-term seizure control.

## 4 Conclusion

The successful treatment of SRSE in a patient with MERRF syndrome underscores the importance of considering VNS as a therapeutic option in refractory cases. VNS therapy proved to have long-term effectiveness in treating epilepsy in a patient with MERRF. This case also emphasizes the need for a multidisciplinary approach and persistence in exploring diverse treatments for SRSE, particularly when no fatal etiology is identified. VNS appears to be a safe and effective adjunctive therapy, capable of improving outcomes in patients with severe and resistant forms of epilepsy.

Legend for video: In this video, we see the patient two weeks after hospital discharge. She can swim independently using a swimming mask in the pool at her house.

## Ethical statement

Ethical review was waived for this study due to its case report nature, and informed consent for the VNS implant was obtained from the patient. We confirm that the approval of an institutional review board was not required for this work. We confirm that we have read the Journal's position on issues involved in ethical publication and affirm that this work is consistent with those guidelines. We confirm that explicit permission for the publication of the video material was included as part of the informed consent process.

## 6 Contributions

VNS Operation: A.K. Helmers, N.G. Margraf. Analysis and interpretation of data: L. Baysal, N.G. Margraf.

Declaration of competing interest: LB received a travel grant from Angelini Pharma. AKH: Consulting for Boston Scientific and Insightec. NGM received a grant from the Health Ministry of Schleswig-Holstein, Germany and travel grants from Eisai Pharma, Angelini Pharma and Jazz Pharma. Lecture honoraria were given by Jazz Pharma, Angelini Pharma, LivaNova and UCB Pharma. Financial support for a patient counselling project was granted by Jazz Pharma, Angelini Pharma, Eisai Pharma, UCB Pharma, LivaNova and Desitin Pharma.

## Funding sources

We would like to thank the Kallsen Family Foundation for supporting our epilepsy research. The support financed LB's research activities. The founders did not exert any influence on the content.

## CRediT authorship contribution statement

**Leyla Baysal:** Writing – review & editing, Writing – original draft, Visualization, Validation, Supervision, Methodology, Investigation, Data curation, Conceptualization. **Sabrina Jobi:** Investigation. **Simone Zimmermann:** Investigation. **Ann-Kristin Helmers:** Investigation. **Nils Gerd Margraf:** Writing – review & editing, Visualization, Validation, Supervision, Software, Resources, Project administration, Investigation, Funding acquisition, Formal analysis, Conceptualization.

## Declaration of competing interest

The authors declare that they have no known competing financial interests or personal relationships that could have appeared to influence the work reported in this paper.
